# Modelling personal temperature exposure using household and outdoor temperature and questionnaire data: implications for epidemiological studies

**DOI:** 10.1016/j.envint.2024.109060

**Published:** 2024-10-09

**Authors:** Xi Xia, Ka Hung Chan, Yue Niu, Cong Liu, Yitong Guo, Kin-Fai Ho, Steve Hung Lam Yim, Baihan Wang, Aiden Doherty, Daniel Avery, Pei Pei, Canqing Yu, Dianjianyi Sun, Jun Lv, Junshi Chen, Liming Li, Peng Wen, Shaowei Wu, Kin Bong Hubert Lam, Haidong Kan, Zhengming Chen, on behalf of the China Kadoorie Biobank Study Group, International Steering Committee, Junshi Chen, Zhengming Chen, Robert Clarke, Rory Collins, Liming Li, Jun Lv, Richard Peto, Robin Walters, International Co-ordinating Centre, Oxford:, Daniel Avery, Maxim Barnard, Derrick Bennett, Ruth Boxall, Ka Hung Chan, Yiping Chen, Zhengming Chen, Charlotte Clarke, Johnathan Clarke, Robert Clarke, Huaidong Du, Geoffrey Ma, Ahmed Edris Mohamed, Hannah Fry, Simon Gilbert, Pek Kei Im, Andri Iona, Maria Kakkoura, Christiana Kartsonaki, Kshitij Kolhe, Hubert Lam, Kuang Lin, James Liu, Mohsen Mazidi, Iona Millwood, Sam Morris, Qunhua Nie, Alfred Pozarickij, Maryam Rahmati, Paul Ryder, Dan Schmidt, Becky Stevens, Iain Turnbull, Robin Walters, Baihan Wang, Lin Wang, Neil Wright, Ling Yang, Xiaoming Yang, Pang Yao, National Co-ordinating Centre, Beijing:, Xiao Han, Can Hou, Qingmei Xia, Chao Liu, Jun Lv, Pei Pei, Dianjianyi Sun, Canqing Yu, Lang Pan, 10 Regional Co-ordinating Centres:, Qingdao CDC, Zengchang Pang, Ruqin Gao, Shanpeng Li, Haiping Duan, Shaojie Wang, Yongmei Liu, Ranran Du, Yajing Zang, Liang Cheng, Xiaocao Tian, Hua Zhang, Yaoming Zhai, Feng Ning, Xiaohui Sun, Feifei Li, Licang CDC, Silu Lv, Junzheng Wang, Wei Hou, Heilongjiang Provincial CDC, Wei Sun, Shichun Yan, Xiaoming Cui, Nangang CDC, Chi Wang, Zhenyuan Wu, Yanjie Li, Quan Kang, Hainan Provincial CDC, Huiming Luo, Tingting Ou, Meilan CDC, Xiangyang Zheng, Zhendong Guo, Shukuan Wu, Yilei Li, Huimei Li, Jiangsu Provincial CDC, Ming Wu, Yonglin Zhou, Jinyi Zhou, Ran Tao, Jie Yang, Jian Su, Suzhou CDC, Fang Liu, Jun Zhang, Yihe Hu, Yan Lu, Liangcai Ma, Aiyu Tang, Shuo Zhang, Jianrong Jin, Jingchao Liu, Guangxi Provincial CDC, Mei Lin, Zhenzhen Lu, Liuzhou CDC, Lifang Zhou, Changping Xie, Jian Lan, Tingping Zhu, Yun Liu, Liuping Wei, Liyuan Zhou, Ningyu Chen, Yulu Qin, Sisi Wang, Sichuan Provincial CDC, Xianping Wu, Ningmei Zhang, Xiaofang Chen, Xiaoyu Chang, Pengzhou CDC, Mingqiang Yuan, Xia Wu, Xiaofang Chen, Wei Jiang, Jiaqiu Liu, Qiang Sun, Gansu Provincial CDC, Faqing Chen, Xiaolan Ren, Caixia Dong, Maiji CDC, Hui Zhang, Enke Mao, Xiaoping Wang, Tao Wang, Xi zhang, Henan Provincial CDC, Kai Kang, Shixian Feng, Huizi Tian, Lei Fan, Huixian CDC, XiaoLin Li, Huarong Sun, Pan He, Xukui Zhang, Zhejiang Provincial CDC, Min Yu, Ruying Hu, Hao Wang, Tongxiang CDC, Xiaoyi Zhang, Yuan Cao, Kaixu Xie, Lingli Chen, Dun Shen, Provincial CDC, Xiaojun Li, Donghui Jin, Li Yin, Huilin Liu, Zhongxi Fu, Liuyang CDC, Xin Xu, Hao Zhang, Jianwei Chen, Yuan Peng, Libo Zhang, Chan Qu

**Affiliations:** 1 Department of Occupational and Environmental Health, School of Public Health, Xi’an Jiaotong University Health Science Center, Xi’an, China; 2 Key Laboratory of Environment and Genes Related to Diseases, Ministry of Education, Xi’an, China; 3 Clinical Trial Service Unit and Epidemiological Studies Unit, Nuffield Department of Population Health, University of Oxford, Oxford, UK; 4 School of Public Health, Shaanxi University of Chinese Medicine, Xi’an, China; 5 The Jockey Club School of Public Health and Primary Care, The Chinese University of Hong Kong, Hong Kong SAR, China; 6 School of Public Health, Key Lab of Public Health Safety of the Ministry of Education and NHC Key Lab of Health Technology Assessment, Fudan University, Shanghai, China; 7 Asian School of the Environment, Nanyang Technological University, Singapore; 8 Lee Kong Chian School of Medicine, Nanyang Technological University, Singapore; 9 Earth Observatory of Singapore, Nanyang Technological University, Singapore; 10 Big Data Institute, Li Ka Shing Centre for Health Information and Discovery, University of Oxford, Oxford, UK; 11 National Institute of Health Research Oxford Biomedical Research Centre, Oxford University Hospital NHS Foundation Trust, John Racliffe Hospital, Oxford, UK; 12 Peking University Center for Public Health and Epidemic Preparedness & Response, Beijing, China; 13 Department of Epidemiology and Biostatistics, School of Public Health, Peking University Health Science Center, Beijing, China; 14 Key Laboratory of Epidemiology of Major Diseases ( Peking University), Ministry of Education, Beijing, China; 15 China National Center For Food Safety Risk Assessment, Beijing, China; 16 Maiji Center for Disease Control and Prevention, Gansu, China; 17 Children’s Hospital of Fudan university, National Center for Children’s Health, Shanghai, China

**Keywords:** climate change, epidemiology, heart rate, temperature, wearables

## Abstract

Non-optimal temperature is a leading risk factor for global disease burden. Most epidemiological studies assessed only outdoor temperature, with important uncertainties on personal exposure misclassification. The CKB-Air study measured personal, household (kitchen and living room), and outdoor temperatures in the summer (MAY-SEP 2017) and winter (NOV 2017-JAN 2018) in 477 participants in China. After data cleaning, ~88,000 person-hours of data were recorded across each microenvironment. Using multivariable linear regression (MLR) and random forest (RF) models, we identified key predictors and constructed personal exposure prediction models. We used generalised additive mixed effect models to examine the relationships of personal and outdoor temperatures with heart rate. The 24-hour mean (SD) personal and outdoor temperatures were 29.2 (3.8) °C and 27.6 (6.4) °C in summer, and 12.0 (4.0) °C and 7.5 (4.2) °C in winter, respectively. The temperatures across microenvironments were strongly correlated (Spearman’s ρ: 0.86-0.92) in summer. In winter, personal temperature was strongly related to household temperatures (ρ: 0.74-0.79) but poorly related to outdoor temperature (ρ: 0.30). RF algorithm identified household and outdoor temperatures and study date as top predictors of personal temperature exposure for both seasons, and heating-related factors were important in winter. The final MLR and RF models incorporating questionnaire and device data performed satisfactorily in predicting personal exposure in both seasons (R^2^_summer_: 0.92; R^2^_winter_: 0.68-0.70). We found consistent U-shaped associations between measured and predicted personal temperature exposures and heart rate (lowest at ~14.5°C), but a weak positive linear association with outdoor temperature. Personal and outdoor temperatures differ substantially winter, but prediction models incorporating household and outdoor temperatures and questionnaire data performed satisfactorily. Exposure misclassification from using outdoor temperature may produce inappropriate epidemiological findings.

## 1 Introduction

Humans have an intricate thermoregulation system that maintains their core body temperature within a relatively narrow range for optimal functionality, regardless of air temperature.^1^ Cold or heat, or “non-optimal temperatures”, challenges the thermoregulation system and could result in a range of potentially harmful physiological and molecular effects.^1–4^ With the worsening climate change and projected rise of thermal stress and temperature extremes, there have been growing concerns about the public health impact of non-optimal temperatures.^5^

Extensive evidence from multi-country time-series and case-crossover studies have shown the acute impact of non-optimal air temperatures on mortality and morbidity, especially for cardio-respiratory diseases.^6–11^ A recent study estimated that non-optimal temperatures accounted for over 5 million deaths in 2019, with the majority occurred in low- and middle-income countries (LMICs).^10^ Notably, the vast majority of temperature-related epidemiological studies used outdoor temperature as a proxy to assess personal exposure. However, most people spend 80-90% of their time indoors,^12,13^ and there exist strong behavioural (e.g., avoidance of outdoors) or infrastructural (e.g., air-conditioning) moderators that could dis-associate personal and outdoor temperatures.^14,15^

In a recent global study based on well-established thermal comfort databases covering 22 countries, a weak-to-moderate relationship was found between indoor and outdoor temperatures (Spearman’s ρ <11.5°C: -0.12; ≥11.5°C: 0.55).^15^ Data on personal exposure have been limited, and mostly conducted in individuals of specific occupations (e.g., outdoor workers) or from high-income countries (HICs).^14,16^ The few existing studies in LMICs reported distinctive personal temperature exposure patterns, but they were restricted to relatively small sample sizes (n ≤ 50 except one) and/ or climate conditions.^17–20^ Furthermore, while personal air pollution exposure prediction models using questionnaire and household measurements data have been developed,^21^ to our knowledge, no studies using the same approach have been published for personal temperature exposure modelling. Importantly, to our knowledge, there is no systematic investigation on the possibly distinctive relationships of personal versus household or outdoor temperatures with health outcomes in the same study sample.

Here, using detailed questionnaire and device data collected in the CKB-Air study in China,^22,23^ we performed a comprehensive investigation aiming to: (i) characterise the relationships between personal, household and outdoor temperatures, (ii) develop personal exposure prediction models, and (iii) compare the associations of temperatures in different microenvironments with heart rate.

## 2 Methods

### 2.1 Study design and population

Details of the design and participant characteristics of the CKB-Air study have been described previously.^22,23^ Briefly, in 2017-2018 the CKB-Air study recruited 477 adults (mean age 58 years, 72% women) from two rural (Gansu, Henan) and one urban (Suzhou) sites using convenient sampling within the China Kadoorie Biobank (CKB),^24^ an ongoing nationwide prospective cohort study of ~512,000 adults recruited from ten areas in China in 2004-2008 (eFigure 1).

In CKB-Air, temperature, relative humidity (RH), and air pollution were measured repeatedly in the warm (May-September 2017; hereafter referred to as ‘summer’) and cool (November 2017-January 2018; ‘winter’) seasons, along with time-activity questionnaires, heart rate measurements, and a household questionnaire (winter only). Overall, 452 and 450 individuals participated in the summer and winter campaigns, respectively, with 37 participants from the summer survey replaced by 35 other eligible CKB participants in the winter due to unavailability (hence total n=477). We have previously shown that participants of the two campaigns had similar baseline characteristics recorded in the original CKB cohort.^22,23^

The study was approved by the Oxford University Tropical Research Ethics Committee, Oxford, UK (Ref: 5109-17) and the institutional review board of Fuwai Hospital, Chinese Academy of Medical Sciences, Beijing, China (Ref: 2018-1038). All participants provided written informed consent upon recruitment.

### 2.2 Temperature measurements

For each participant, in each season we collected up to 120 consecutive hours (covering weekdays and weekend) of temperature data (along with other environmental exposures) at 1-min resolution across the personal, household (kitchen and living room), and outdoor environments (except for those who only participated in one season). Due to logistical constraints, the measurements were conducted in batches of participants by study areas, and the time period of measurements for each area was chosen to closely align the climate conditions of the summer and winter based on historical data.^22,23^ For each season, all measurements within each area were completed within five weeks to ensure comparability between batches of participants. For the personal and household measurements, we used an internationally validated lightweight monitor, PATS (Particle and Temperature Sensor; Berkeley Air Monitoring Group, CA, USA), which measures temperature and relative humidity using an individually manufacturer-calibrated SHT21 sensor (Sensirion AG, Zurich, Switzerland; accuracy ±0.3°C; range: -40.0°C to 123.8°C) designed to measure the temperature of the air stream brought into the device by a micro-fan. Participants were instructed to always carry the PATS, with a cross-body harness or waist belt on top of their clothing, except during bathing or sleeping, when the device should be placed within 1m from the participants. The two household PATSs were placed in undisturbed locations in the kitchen and living room, respectively, at about 1.5m above the ground and ≥1m away from any doors or other wall-openings. Outdoor temperature was measured by two NAS-AF100 (Sapiens Environmental Technology, Hong Kong, China), placed at a central location of the community, at least two stories above the ground, for the whole survey period in each community. For each participant, we assigned the corresponding NAS-AF100 data as their outdoor temperature exposure during their personal measurement periods. As for the 10 participants in the summer and 74 in the winter with missing NAS-AF100 data, we used the regional temperature data derived from the well-established 5^th^ generation European centre for Medium-Range Weather Forecasts reanalysis database for global climate and weather (ERA5) based on the geolocation of the centroid of the study community.^25^

### 2.3 Household and time-activity questionnaires

Electronic questionnaires were administered by trained fieldworkers using bespoke software with built-in error and logic checks. The household questionnaire (administered in the winter) assessed personal (e.g., age, sex, household income) and household (e.g., heating fuel use, ventilation) characteristics in the past year. The time-activity questionnaire (up to four times per participant), captured 24-hour recall of participants’ activity (12 categories) and its location (10 categories) and duration (5-minute resolution) on one weekday and one weekend-day during the temperature monitoring period per season (eTable 1), from which participants’ season-specific average percentage of time spent indoors (i.e., kitchen, living room, bedroom, home [unspecific], work/public [indoors]) was ascertained.

### 2.4 Heart rate measurements

Repeated heart rate measurements (in beat-per-minute, bpm) were taken by trained health workers on one weekday and one weekend-day (twice each day), using a medical-grade Onyx Vantage 9590 Finger Pulse Oximeter (Nonin Medical Inc., MN, USA; range: 18 to 321 bpm; accuracy: ±3bpm), after the completion of the time-activity questionnaire, when participants were seated away from any source of air pollution for about 10 minutes. The oximeter was operated as per the manufacturer’s manual and heart rate measurements (in duplicates) were taken from the same index finger. The present study used the average across four measurements recorded on one weekday and one weekend-day per season.

### 2.5 Data processing

Modelled on a previously described data cleaning protocol,^22,23^ we processed the time-resolved temperature data by i) removing data collected during the first and last hour of each measurement period, when participants’ behaviour may be affected by study procedures, ii) removing participants with <24-hour of effective data (mostly due to battery failure), iii) restricting the analysis with sufficient effective data in all three PATS_+_ monitors, iv) removing participants with potentially faulty devices recording unrealistically stable temperature (intra-period [i.e. up to 120-hour] variability at 5-min resolution <2°C), and v) incorporating the corresponding NAS-AF100 and ERA5 outdoor temperature data (eFigure 2). We then aligned the household and time-activity questionnaire data for each participant with processed device data (n_summer_=391; n_winter_=403; n_both_seasons_=354) for the main analyses (i.e. those with no questionnaire data were excluded), resulting in a total of 87,238 person-hours of data each at the personal, kitchen, and living room, and 88,069 person-hours of data at the outdoor level (eTable 2). While the aim of the temperature measurements were to ascertain 24-hour average exposure from up to 120 consecutive hours of monitoring per season, the participants remained after the above data cleaning procedures recorded mean (SD) ranging from 103 (20.4) to 114 (17.4) hours of temperature across the four microenvironments (eTable 2), with only up to one (0.25%) participant recording <48 hours of data in one of the microenvironments. Additionally, we excluded participants without heart rate data for health-related analyses (n_summer_ =390; n_winter_=394).

### 2.6 Statistical analysis

We examined the season-specific age, sex, and area-adjusted mean (95 % confidence intervals [CI]) daily temperature across personal, household, and outdoor environments by participant characteristics. For each location, we calculated the intra-period temperature variability as maximum minus minimal daily mean temperature (up to five days per season per participant); and inter-season temperature variability as mean daily temperature in summer minus that in winter. We explored the relationships between season-specific and weighted annual average temperatures recorded across locations using linear regression and Spearman’s rank correlation (ρ).

We applied a Boruta algorithm, a random forest (RF) based machine learning approach,^26^ to identify relevant predictors of personal temperature by season, out of a list of 21 variables selected based on prior knowledge, with a p-value threshold of 0.01 and a maximal number of 50,000 runs. Specifically, the algorithm repeatedly generate shadow features by shuffling the values of the predictors in a duplicated dataset and train a RF classifier to compute shadow feature importance (as the maximum Z-score of mean decrease accuracy), which is then compared against the corresponding Z-score generated from the random forest model trained on the original data, with features showing statistically significantly higher Z-score identified as important. We presented boxplots of the feature importance measure (i.e., Z-scores of mean decrease accuracy) in descending order. The direction of association of each feature with personal temperature exposure was evaluated using multivariable linear regression (MLR) adjusted for age, sex, and area. A random-number variable was included in the model as a negative control to detect potential biases (e.g. inflation of importance for continuous predictors).

In developing the season-specific, mean daily personal temperature exposure (i.e., one average value per participant per season) prediction models, we compared two well-established approaches with complementary advantages, namely MLR and RF algorithm. Based on the Boruta-identified features, eight sets of questionnaire and device variables (with overlaps) were evaluated for various model performance indicators (R-squared [R^2^], root mean squared error [RSME], normalised RMSE [nRMSE], and Akaike information criterion [AIC]) (Table 1). The number of variables and decision trees in the RF models were selected automatically based on the maximum R^2^, and the corresponding RMSE and nRMSE were reported. Further 10-fold cross-validation (CV) was performed to assess the robustness of the MLR and RF models, and the CV-R^2^ and CV-RMSE were reported, along with graphical illustration of the linear fittings and Bland-Altman plots^27^ between predicted and measured personal temperatures from the final models. Sensitivity analyses were conducted: (i) using linear mixed effect (LME) regression to predict repeated measurements of 24-hour mean temperature, with participant ID included as a random effect to parametrize the auto-correlation between repeated measurements; and (ii) by excluding participants with ERA5-modelled outdoor temperature data.

We used generalised additive mixed models (GAMM) to investigate the exposure-response relationships of measured and predicted (by different MLR and RF models) personal temperature exposures and measured outdoor temperature with average heart rate by season, adjusting for age, sex, region, education, occupation, income, temporal trend, and natural cubic splines with 3 degrees of freedom for fine particulate matter (PM_2.5_) and RH.

All analyses were conducted in R version 4.3.1, and we used the *Boruta, lm, randomForest, gamm4*, and *lme4* packages for the aforementioned main analyses.

## 3 Results

### 3.1 Participant characteristics and temperature exposures

Participant characteristics by measurement campaign attended are shown in eTable 3, with no significant difference by season, except ~5% higher self-reported time spent indoors in winter (mean [SD]=85.5 [12.0]%) than summer (90.1 [10.0]%). Importantly, participants spent 96.4% of their indoor time at home (i.e., 82.5% of all time were spent at home). Across microenvironments, outdoor temperature was the lowest in both seasons (mean_summer_= 27.6°C; mean_winter_= 7.5°C), with larger differences from personal (~4.5°C) or household (~3.1°C) temperatures in winter (Table 2). For both seasons, temperature variability was the highest for outdoor environments (5.6-6.2°C), followed by personal (3.0-4.3°C) and household environments (2.0-2.6°C), with personal temperature variability showing a particular rise (43%) from summer to winter (eFigure 3). The inter-season temperature variability appeared similar across household and outdoor environments (18.6-20.1°C), but somewhat lower at personal level (17.0°C).

### 3.2 Relationships between temperatures across microenvironments

**Figure 1:**
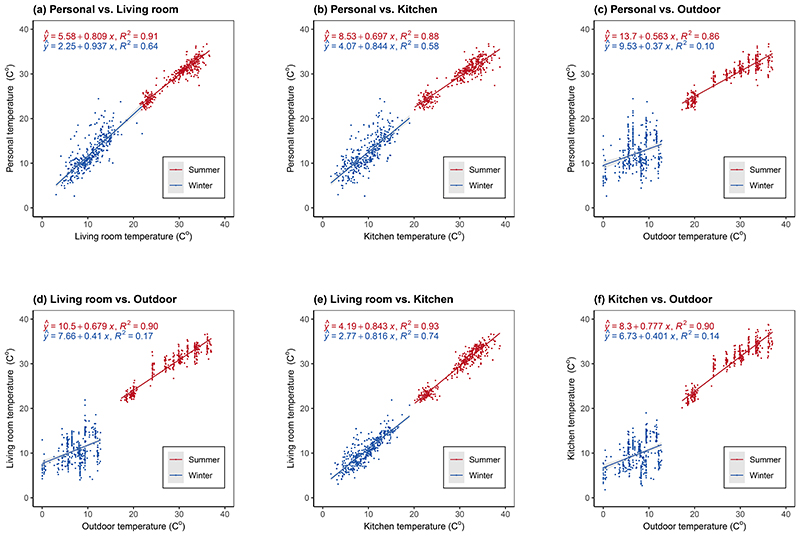
Linear regression fitting plots of measured temperature across locations by season Shaded area around regression lines indicate 95% confidence intervals

Temperature across personal, household, and outdoor locations were strongly correlated in summer (Spearman’s ρ range: 0.86-0.92; eFigure 4), but in winter personal and household temperatures were markedly better correlated with each other (0.74-0.85) than with outdoor temperature (0.30-0.35). For each 1 °C lower outdoor temperature in winter, personal temperature only drops by 0.39°C (Figure 1c), compared to the corresponding values of 0.84-0.94°C with respect to each 1 °C lower household temperature (Figures 1a and 1b). While both household and outdoor temperatures were good predictors of personal temperature in the summer (R^2^ range 0.86-0.91), in winter household temperatures (R^2^ range 0.58-0.64) performed much better than outdoor temperature (R^2^=0.11) (Figure 1). However, when using weighted annual average temperature (eFigure 5), the performance of outdoor temperature (R^2^ = 0.70) in predicting personal exposure was comparable to that of kitchen temperature (0.69), but still weaker than that of living room temperature (0.88).

### 3.3 Boruta feature selection and personal exposure prediction models

**Figure 2:**
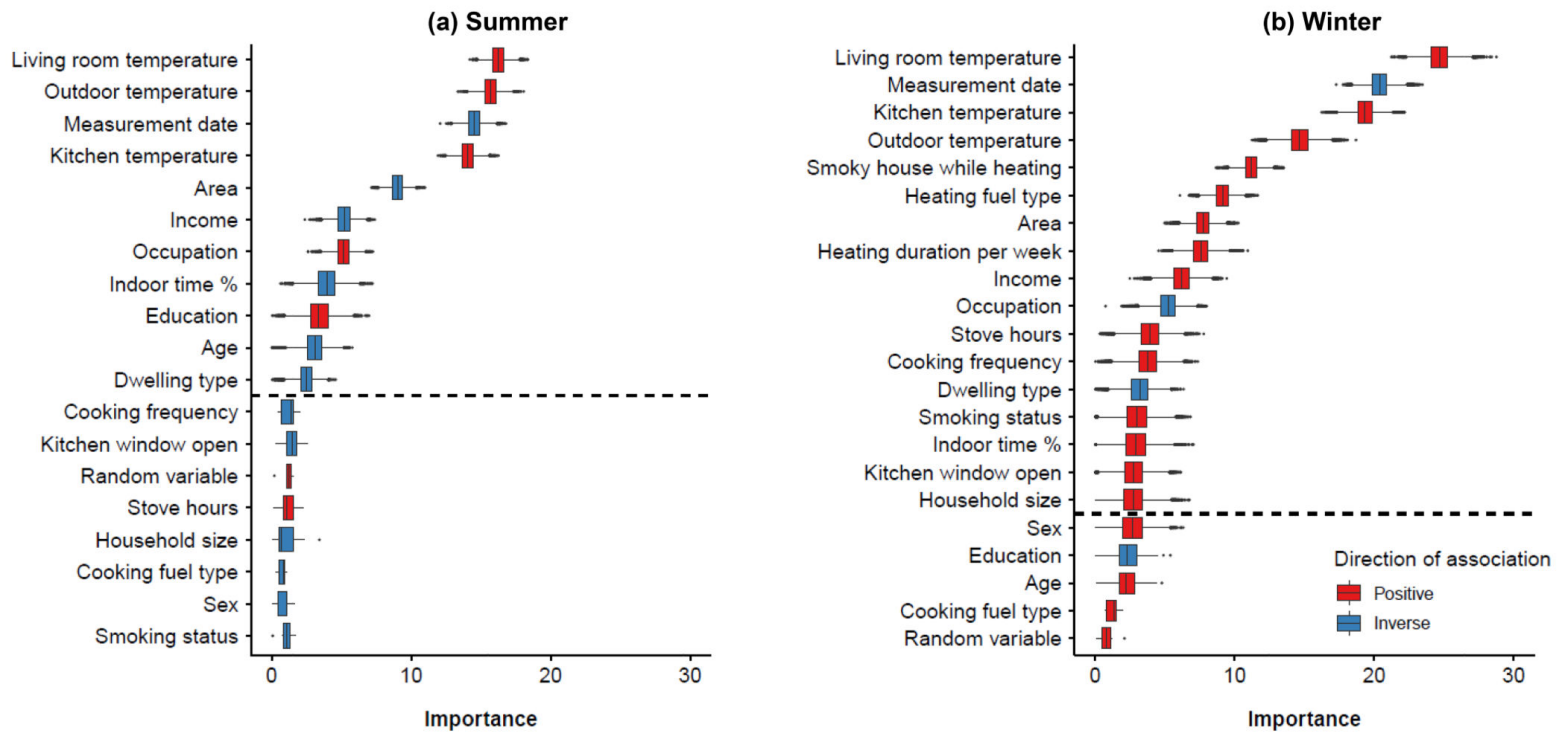
Ranked importance of variables identified in Boruta feature selection for personal temperature exposure by season Features above the horizontal dashed line indicate meets the pre-specified criteria of important features. The direction of association was ascertained from multivariable linear regression of each feature on measured personal temperature, adjusting for age, sex, and study area where appropriate.

The Boruta algorithm identified 11 and 17 predictors of personal temperature exposure in summer and winter, respectively, and the random-number was found to be irrelevant as expected (Figure 2). Notably, household and outdoor temperatures and study date were consistently identified as the most important features in both seasons, followed by (in no particular order) study area, socioeconomic indicators, heating-related exposures (winter only), and percentage time spent indoors (higher rank in summer than winter).

Across the prediction models, the CV-R^2^ were larger in summer (MLR: 0.38-0.92; RF: 0.88-0.92) than winter (MLR: 0.42-0.69; RF: 0.60-0.70), with RF outperforming MLR in general (Table 3). Adding selected questionnaire data to the basic model resulted in significant improvement in the summer MLR model, but minor improvements in the winter MLR or RF models in both seasons. The most significant single-step improvement occurred upon adding living room temperature to the basic model (CV-R^2^_MLR_summer_=0.92; CV-R^2^_MLR_winter_=0.67; CV-R^2^_RF_summer_=0.90; CV-R^2^_RF_winter_=0.67). Further addition of temperature in other microenvironments or questionnaire variables resulted in modest improvements for both models, with slightly higher CV-R^2^ in the final models including all Boruta-selected features (summer: 0.92 for both MLR and RF; winter: 0.68 for MLR and 0.70 for RF). Sensitivity analysis showed that LME models performed considerably worse than the MLR and RF models (eTable 4). The performance of the MLR and RF models based on PATS and NAS-F100 data (i.e. excluding ERA5-modelled outdoor temperature) was broadly consistent with the main models, with only modest decline in the winter models with significantly less data points (eTable 5).

**Figure 3:**
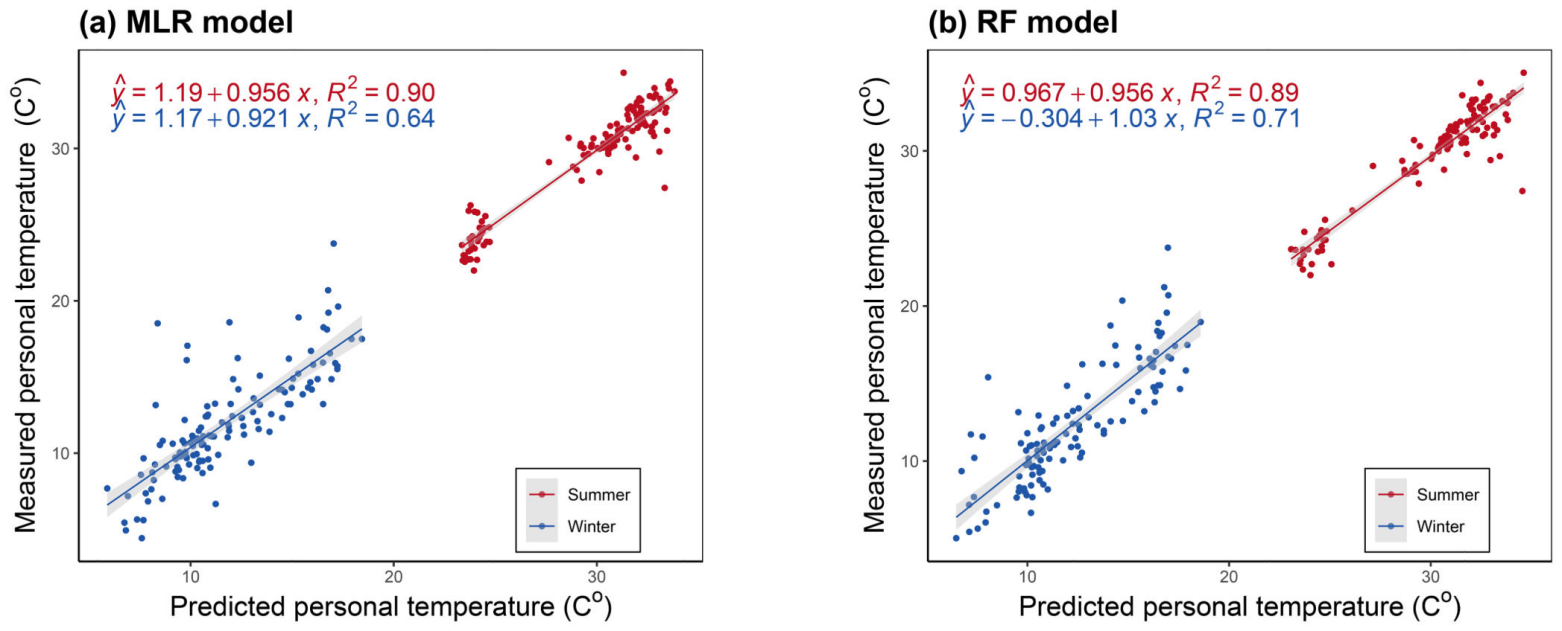
Linear regression fitting plots of measured and predicted personal temperature from the final multivariable linear regression and random forest models by season Shaded area around regression lines indicate 95% confidence intervals. Abbreviations: MLR = multivariable linear regression; RF = random forest.

**Figure 4:**
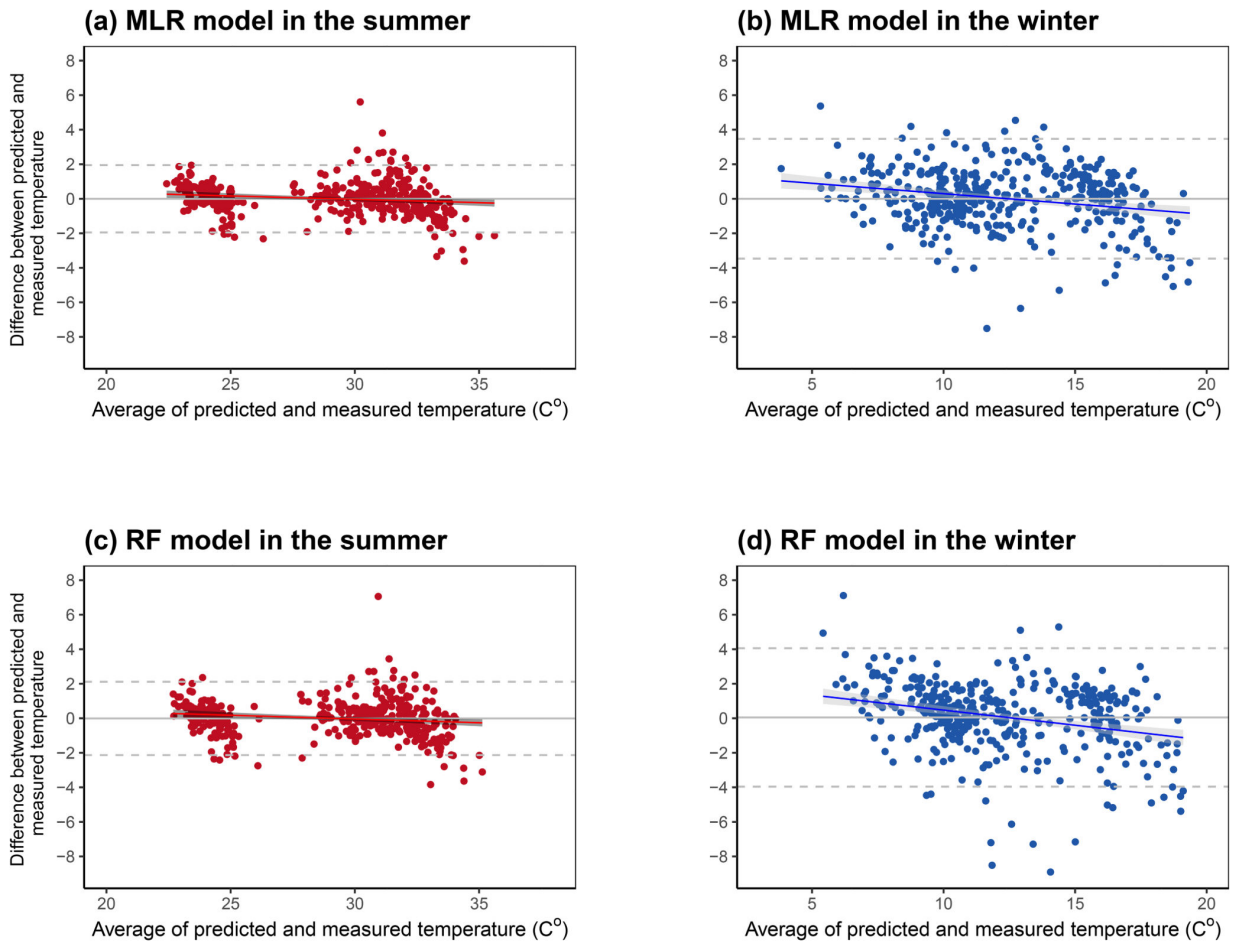
Bland-Altman plots of measured and predicted personal temperature from the final multivariable linear regression and random forest models by season Regression lines and 95% confidence intervals (shaded area) were obtained from linear regression of the difference between measured and predicted personal temperature on the average of the two. Grey horizontal dashed lines represent the upper and lower limits of agreement (± 1.96SD). Abbreviations: MLR = multivariable linear regression; RF = random forest.

The linear fitting of the measured and predicted personal temperature from the final MLR and RF models showed satisfactory performance with values closely scattered around the identity line for both seasons, even though the winter models have somewhat smaller R^2^ (Figure 3). Consistently, Bland-Atman plots show satisfactory validity with approximately zero mean difference between predicted and measured personal temperature for both models in both seasons (Figure 4), although the models in winter had a slight tendency of overestimation at lower temperature and underestimation at higher temperature. The 95% limits of agreement was about ±2°C in summer and ±4°C in winter for both models.

### 3.4 Exposure-response relationship between temperature and heart rate

**Figure 5:**
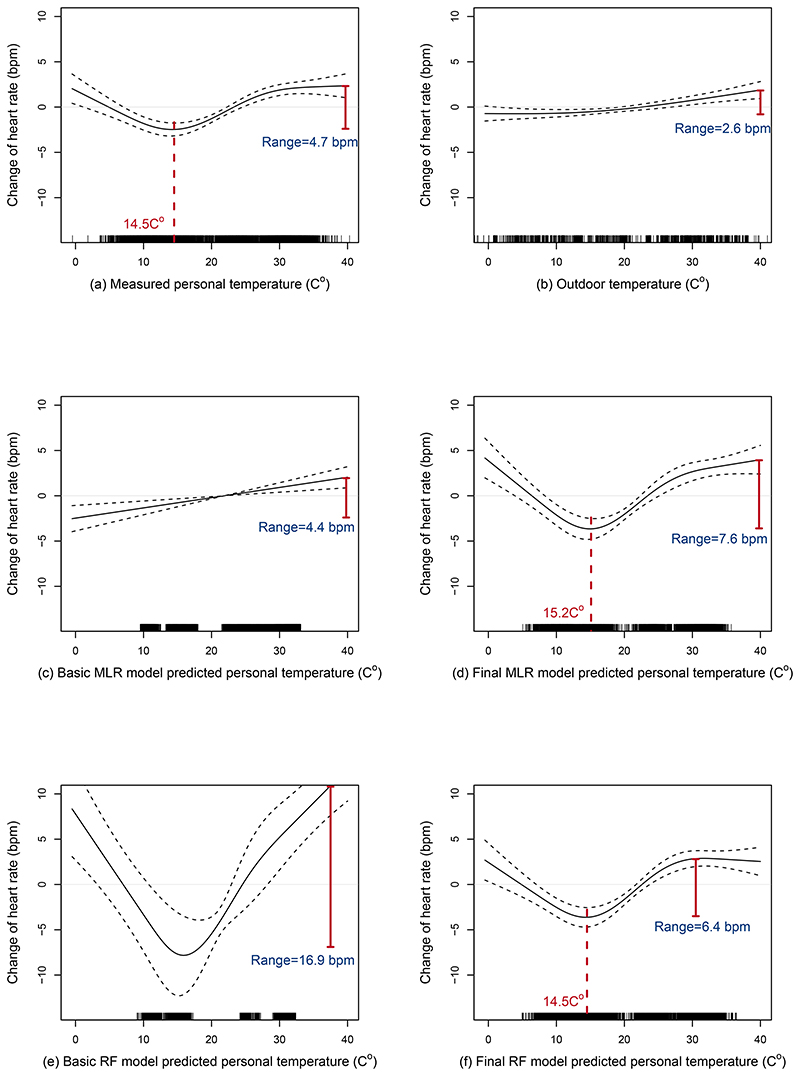
Exposure-response functions between measured and predicted temperature and heart rate Change of hear rate (beat-per-minute) was derived from generalized additive mixed models adjusting for age, sex, region, education, occupation, income, temporal trend, and natural cubic splines with 3 df for fine particulate matter and relative humiditiy. Dashed lines around the exposure-response curve represent 95% confidence intervals. Vertical purple dashed lines indicate the point of lowest heart rate and the corresponding temperature in plots A, D, and F. The indicated range of bpm refers to the difference between the highest mean heart rate estimate from the high temperature end compared to the point of lowest mean heart rate of the exposure-response function. Black vertical lines at the bottom indicate the distribution of exposure data across the temperature range on the x-axis.

An approximately U-shaped relationship was found between measured personal temperature and mean heart rate, with the lowest heart rate appeared at 14.5°C and elevated heart rate at both the low and high temperature ends (Figure 5a). Statistically significant elevated heart rate emerged below 5.3°C and above 23.5°C, and the peak-to-trough heart rate range at high temperature (HR_peak-to-trough_) was 4.7 bpm. Interestingly, outdoor temperature (Figure 5b) and basic-MLR predicted personal temperature (Figure 5c) showed a weak positive linear relationship with heart rate, in contrast to the U-shaped relationships between final MLR and RF predicted personal temperatures and HR (Figures 5d and 5f). The predicted personal temperatures with the lowest heart rate (14.5-15.2°C) were highly consistent with the measured data (14.5 °C), although the HR_peak-to-trough_ were somewhat larger (MLR: 7.6 bpm; RF: 6.4 bpm).

## 4 Discussion

In a reasonably sized study sample from urban and rural China, we depicted a strong agreement between personal, household, and outdoor temperatures in summer, but poor agreement between outdoor and personal or household temperatures in winter, while the personal-household temperature relationship remained moderate-to-strong in both seasons. We developed prediction models that performed better in summer than winter, with the RF models modestly outperforming the MLR models. We found U-shaped associations between measured and modelled personal temperatures and heart rate, but a weak positive linear association with outdoor temperature.

### 4.1 Relationships between temperatures across microenvironments

In building science there is a common interest in effectively maintaining a narrow temperature range of thermal comfort in residential buildings, regardless of outdoor temperature. This has fuelled ample investigations on the relationship between outdoor and indoor temperatures.^15^ The largest building science study to date involved ~68,000 field measurements in 86 cities across 22 countries^15^ and reported a weak-to-moderate association (Spearman’s ρ <11.5°C: -0.12; ≥11.5°C: 0.55) between indoor and outdoor temperatures, with 0.33°C and 0.16°C increase in indoor temperature per 1°C higher outdoor temperature in summer and winter, respectively.^15^ On the other hand, studies on personal temperature exposure have been predominantly conducted in HICs,concerned primarily occupational exposure in urban settings, and focused on heat instead of a broader temperature ranges.^14,16,28–31^ Of the limited studies on personal temperature measurements in LMICs, most were conducted in warm, rural regions of India (generally >18°C in the cool season) and involved relatively short-term (24-hour) monitoring in limited sample sizes, with only two studies having repeated measurements across seasons.^18–20^ These studies found moderate-to-strong agreement between personal or household temperatures and outdoor temperature in general, but somewhat weaker associations in the cool season.^18–20^

Compared to previous personal measurement studies involving largely rural residents in similar LMIC settings,^18–20^ we employed similar approaches to collect time-resolved temperature data using well-established environmental sensors. As an novel contribution to the literature, in the CKB-Air we captured 6-10 times greater amount of data (in person-hours) across a broader outdoor temperature range (mean daily temperature range: -3.9°C to 42.6°C versus >18°C in previous studies) and across the personal, household, and outdoor environments, while most previous studies only compared personal versus outdoor temperatures.^18,19^ In summer, we found a considerably stronger agreement between personal and household or outdoor temperatures than previous studies, which may reflect differences in housing infrastructure (e.g. housing materials) and temperature-related behaviour (e.g. avoidance of heat) between populations. In summer, the most powerful moderator of personal versus outdoor temperature would be air-conditioning, which was extremely rare in our study population (based on field observations and the high household temperatures recorded). While most previous studies did not cover colder temperatures, in CKB-Air we found weaker correlation between personal and outdoor temperatures in winter, but better agreement between personal and household temperatures. Importantly, currently cold temperatures are associated with much more substantial disease burden than heat.^10^ Our findings highlight the intricacies of personal temperature exposure, which was markedly more strongly related to household than outdoor temperature in winter in a population with reasonable household heating coverage. Nonetheless, in CKB-Air the relatively low household temperatures (12-14°C indoors vs 7-9°C outdoors) suggest that the existing heating infrastructure or intensity of usage were weaker than in more affluent populations with better central heating coverage, where the correlation between personal and outdoor temperatures could be even lower.^15^ Behavioural factors such as percentage of time spent indoors, space heating, and cooking duration appeared important predictors of personal temperature in winter as well. Notably, when we examined weighted annual averages (as opposed to seasonal averages), the personal-outdoor temperature agreement was reasonably strong (R^2^ = 0.70; □: 0.41°C), as the annual average estimation was dominated by warm temperatures in summer. While this may not be quantitatively generalisable to other populations with different behavioural, housing, and climate conditions, the discrepancies of personal-outdoor temperature agreement underlies how exposure misclassification may vary by timeframe of investigation. By taking a longer-term average, misclassification over a short-term (e.g., in days) could be diluted and thus less impact on epidemiological estimations. Our findings also underscore the need to consider the possibility of differential misclassification in short-term epidemiological analysis on temperature covering different seasons, and future studies should consider season-specific analysis, or, if data is available, adjustment for adaptation factors that could influence the personal-outdoor temperature relationship.

### 4.2 Personal temperature exposure prediction models

To our knowledge, although one previous study of 50 individuals in peri-urban India have depicted predictors of personal temperature exposure,^18^ this is the first study to focus on developing personal exposure prediction models for epidemiological analysis. The Indian study focused on diurnal and daytime versus night-time temperatures, collecting repeated short-term (24-hour) measurements across seasons, but indoor temperature was not monitored,^18^ whereas our study focused on longer-term daily mean temperature across weekdays and weekends and multiple microenvironments. Of the comparatively limited range of participant and environmental characteristics assessed, using LME, they found personal temperature to be positively associated with outdoor temperature, asbestos sheet roof (versus tiles/ grass), and household income and inversely associated with residential or personal altitude and bedroom ceiling height.^18^ Using a well-established machine learning algorithm, we identified features well-expected to be useful predictors of personal temperature exposure, particularly measured household and outdoor temperatures and study date. Generally speaking, the direction of association between predictors and personal temperature shows clear face validity. For example, three heating-related variables (i.e., smoky house while heating, heating fuel type, and heating duration per week) were found to be associated with higher personal temperature exposure in winter; and greater percentage of time spent indoors or higher income was associated with lower personal temperature in summer, but higher in winter.

With up to 120 consecutive hours of data per participant per season, we depicted a previously under-explored gradient of inter-day temperature variability (outdoor > personal > household) and a particular rise of personal temperature variability in winter. In summer, when temperature across locations were consistently high and temperature variability was relatively low, the MLR and RF models had better performance than in winter, when the temperature variability was significantly higher. It is worth-noting that the nRMSEs were more comparable across seasons (while RMSEs were 90-100% greater in winter compared to summer), suggesting that the larger temperature variability in winter was an important challenge in personal temperature exposure prediction.

Among the prediction models, the RF models generally outperformed MLR, indicating possible non-linearity or interaction between predictors and personal exposure, particularly when comparing the models using questionnaire-only data. For both approaches, adding measured living room temperature data resulted in the most obvious improvement in model performance. This is highly consistent with the participants’ high percentage of time spent indoors at home (82.5%), where the living room is the primary or representative microenvironment in terms of temperature exposure. Our findings highlight the potential value of collecting living room temperature in future epidemiological studies, which is highly feasible at scale compared to measuring personal temperature exposure.

The MLR models also outperformed the LME models, indicating that, where data are available, a longer-term average daily temperature across multiple days within a season is more predictable than individual 24-hour mean temperature even after accounting for auto-correlation in LME models. This is consistent with the observation of stronger associations between annual weighted average temperatures across locations than season-specific averages, as averages over a longer timeframe can partially account for the noise from exposure variability.

### 4.3 Exposure-response relationship between temperature and heart rate

The vast majority of the existing temperature-related epidemiological studies used outdoor temperature, either from fixed-site monitoring stations or spatiotemporal models, as a personal exposure proxy.^6–11^ Previous studies on personal temperature exposure rarely measured objective health indicators, but indoor temperature was found to be more strongly related to thermal sensation vote (Spearman’s ρ: 0.42-0.46), a widely utilised self-reported score of thermal comfort, than outdoor temperature (ρ: -0.07-0.09).^15^ In this study, we reported a novel in-sample comparison of epidemiological associations with measured and predicted personal temperatures and outdoor temperature in a LMICs setting. We chose heart rate as an easy-to-measure outcome with relatively well-established relationship with temperature, whereby both cold and heat could increase heart rate with increased thermoregulatory demand (e.g., shivering for cold; increased blood flow to skin for heat dissipation).^4,32^ We found consistent U-shaped associations between measured and predicted (from the final models) personal temperatures and heart rate (although with some quantitative differences), but a weak positive association with outdoor temperature. The stark discrepancies between the shapes of association found for personal versus outdoor temperatures substantiate prevailing concerns about exposure misclassification from relying on outdoor temperature data, at least in the intra-seasonal association examined in this setting. Even if personal exposure data, either from direct measurements or predictive models, are not available, future epidemiological studies should carefully evaluate and discuss the potential extent and implications of misclassification from using outdoor temperature, which could vary widely by populations. Furthermore, with the strong agreement of the epidemiological relationships of predicted (from household temperature and behavioural and housing factors) and measured personal temperatures with heart rate, our study highlights housing and behavioural factors as key areas of intervention to shield people from the harm of non-optimal outdoor temperatures.

### 4.4 Strengths and limitations

The key strength of this study lies in the comparatively large and diverse sample, the richness of data collected via repeated (seasonal), parallel measurement of personal, household, and outdoor temperatures and time-activity questionnaire, and the in-sample evaluation of the discrepancies of epidemiological associations with different sources of temperature data. There are also limitations warranting discussion. First, as reported previously, although the self-reported wear time of the PATS was high (>80% of awake time),^23^ we lacked objective validation data. Similarly, the time-activity questionnaire is subject to recall error and bias and was not validated (with e.g., wearable camera). However, previous studies in LMIC settings have shown that, although time-activity questionnaire has risk of misclassification at high time-resolution (e.g. by minutes), it performs reasonably well in capturing longer-term daily or weekly behavioural patterns.^33^ Furthermore, as the CKB-Air was originally advertised as a study of air pollution, with the the generic nature of the time-activity questionnaire, the recall error is unlikely to be systematically associated with temperature exposure and will be incorporated as noise that is reflected in the uncertainty of the statistical analyses presented. Where possible, future studies would be benefited from in-sample validation of time-activity questionnaire. Second, despite the use of in-sample CV in evaluating model performance (and intrinsic advantages of RF models), the findings from the present study, especially the specific ranks of predictors of personal temperature or the shape and strength of the temperature-heart rate relationship, would not be generalizable to other settings with different climate conditions and infrastructural factors (e.g., air-conditioning, central heating). While the key messages on the importance of personal and household temperatures and the possible impact of exposure misclassification on epidemiological analysis should still be relevant to most settings, further studies are needed to depict and validate our findings in diverse populations. Third, personal thermal sensation is subject to not only air temperature but also RH, radiant heat, and wind speed,^34^ but we focused only on air temperature for the feasibility of obtaining reliable measurements that can be more readily applied to large-scale epidemiological studies. Future studies using more complex, multi-modal sensors are needed to explore the feasibility of incorporating other thermal sensation factors in epidemiological studies. Fourth, due to feasibility concerns, the heart rate measurements were only taken at two time points per season instead of monitoring continuously along with temperature exposure. Heart rate is known to exhibit circadian variations with a relatively stable level during daytime (08:00-20:00) and lower levels during night-time and sleep.^35^ Since all measurements were taken at similar daytime across participants, the use of period-specific averages of limited measurements to represent “usual” daytime heart rate should not be subject to significant bias, and the error should not be related to temperature exposure. However, the lack of time-resolved heart rate data prevented us from investigating previously reported time-lagged associations^36^ or the associations of other important temperature metrics (e.g. night-time temperature) with time-specific heart rates.^37,38^

### 4.5 Conclusions

In a comparatively large and diverse sample of Chinese adults with limited air-conditioning or heating, we found strong agreement of temperature measured across personal, household, and outdoor environments in the summer, and personal temperature was moderately correlated with household temperature, but poorly related to outdoor temperature in the winter. We demonstrated the feasibility and value of developing personal temperature exposure prediction models by incorporating household and outdoor temperatures with household and time-activity questionnaire data, showing satisfactory model performance in both seasons. We found important qualitative deviation in the shape of exposure-response relationships of personal versus outdoor temperature with heart rate, substantiating prevailing concerns about exposure misclassification from using outdoor temperature. Future epidemiological studies could leverage the potential of the increasingly popular and reliable household environmental sensors in environmental monitoring to enhance personal exposure estimation and, thus, improve our understanding of the health impact of temperature.

## Supplementary Material

Supplementary file

Supplementary material

## Figures and Tables

**Table 1:** Specifications of models

Models	Variables
Basic	Age, Sex, Measurement date (as a natural cubic smooth function for the first date of measurement with 3 degrees of freedom)
Basic+Questionnaire*	Summer: Basic variables + Area, Income, Occupation, Indoor time %, Education, Dwelling type Winter: Basic variables + Smoky house while heating, Heating fuel type, Area, Heating duration per week, Income, Occupation, Stove hours, Cooking frequency, Dwelling type, Smoking status, Indoor time %, Kitchen window open, Household size
Basic+Outdoor	Basic variables + Outdoor temperature
Basic+Living room	Basic variables + Living room temperature
Basic+Kitchen	Basic variables + Kitchen temperature
Basic+Outdoor+Living room	Basic variables + Outdoor temperature + Living room temperature
Basic+Outdoor+Kitchen	Basic variables + Outdoor temperature + Kitchen temperature
Basic+Outdoor+Living room+Kitchen	Basic variables + Outdoor temperature + Living room temperature + Kitchen temperature
Basic+Questionnaire+Outdoor+Living room+Kitchen	Basic variables + Questionnaire variables + Outdoor temperature + Living room temperature + Kitchen temperature

* Different predictors were included in the summer and winter models as heating-related variables were deemed irrelevant to summer exposure.

**Table 2:** Age, sex- and area-adjusted seasonal mean (standard deviation) temperature (°C) by selected characteristics

Characteristics	Summer (May 2017 – Sep 2017)					Winter (Nov 2017 – Jan 2018)			
	Personal	Kitchen	Living room	Outdoor		Personal	Kitchen	Living room	Outdoor
Overall	29.2 (3.9)	29.7 (5.1)	29.2 (4.4)	27.6 (6.4)		12.0 (4.0)	9.6 (3.5)	10.6 (3.3)	7.5 (4.2)
Age, mean (SD)									
* <65 years*	29.0 (2.4)	29.4 (2.6)	28.8 (2.3)	27.1 (3.6)		12.1 (3.8)	9.6 (3.1)	10.7 (3.0)	7.3 (5.0)
* ≥65 years*	28.8 (4.8)	29.0 (5.2)	28.9 (4.6)	26.8 (7.0)		12.0 (7.5)	9.3 (6.1)	10.5 (5.9)	7.6 (9.8)
Sex									
* Female*	29.0 (2.2)	29.4 (2.4)	29.0 (2.1)	27.2 (3.2)		12.2 (3.5)	9.9 (2.9)	10.8 (2.7)	7.7 (4.5)
* Male*	28.9 (3.7)	29.2 (4.1)	28.6 (3.6)	26.9 (5.5)		12.0 (6.0)	9.2 (4.9)	10.6 (4.7)	7.0 (7.7)
Education									
* No formal*	29.1 (4.6)	29.6 (5.0)	29.1 (4.4)	27.5 (6.8)		12.0 (7.5)	9.3 (6.2)	10.2 (5.9)	7.3 (9.8)
* Primary or middle school*	28.8 (3.5)	29.2 (3.8)	28.8 (3.3)	26.9 (5.1)		12.0 (5.4)	9.6 (4.4)	10.4 (4.2)	7.3 (7.0)
* High-school or above*	28.9 (3.2)	29.3 (3.5)	28.7 (3.1)	27.0 (4.7)		12.2 (5.1)	9.6 (4.1)	11.0 (4.0)	7.4 (6.6)
Occupation									
* Agriculture & related workers*	29.1 (4.1)	29.6 (4.5)	29.0 (4.0)	27.6 (6.1)		12.3 (6.8)	9.9 (5.5)	10.6 (5.3)	7.2 (8.8)
* Factory worker*	28.6 (8.7)	28.3 (9.5)	28.5 (8.4)	26.2 (12.9)		11.1 (14.9)	8.6 (12.1)	9.8 (11.7)	7.1 (19.3)
* Self/un-employed or other*	29.0 (5.8)	29.3 (6.2)	29.0 (5.5)	26.6 (8.5)		11.8 (9.7)	8.8 (7.9)	10.7 (7.6)	7.2 (12.6)
Income, yuan/ year									
* <35,000 yuan*	29.3 (4.1)	29.8 (4.4)	29.3 (3.9)	27.7 (6.1)		11.9 (6.5)	9.7 (5.3)	10.7 (5.1)	7.9 (8.3)
* 35,000-74,999 yuan*	28.6 (3.7)	29.0 (4.1)	28.3 (3.6)	26.7 (5.6)		12.2 (6.0)	9.7 (4.9)	10.9 (4.7)	7.6 (7.8)
* ≥75,000 yuan*	28.8 (5.1)	29.1 (5.5)	28.8 (4.8)	26.8 (7.5)		12.1 (8.3)	9.1 (6.7)	10.5 (6.5)	6.6 (10.7)
Dwelling type									
* Apartment*	29.1 (9.9)	31.0 (10.6)	29.9 (9.4)	30.1 (14.2)		14.2 (14.9)	12.4 (12)	13.0 (11.6)	9.3 (19.4)
* House*	28.9 (2.2)	29.3 (2.3)	28.8 (2.1)	27.0 (3.2)		12.0 (3.5)	9.4 (2.8)	10.6 (2.7)	7.3 (4.5)
Area									
* Gansu*	24.1 (3.6)	22.7 (3.9)	23.1 (3.5)	19.4 (5.3)		15.8 (5.7)	13.1 (4.6)	14.0 (4.4)	8.4 (7.3)
* Henan*	30.5 (3.4)	31.5 (3.7)	30.7 (3.3)	28.3 (5.0)		10.1 (5.4)	7.3 (4.4)	9.0 (4.3)	8.7 (7.0)
* Suzhou*	32.2 (3.3)	33.7 (3.6)	32.7 (3.2)	33.5 (4.9)		10.3 (5.4)	8.2 (4.4)	9.1 (4.2)	5.0 (7.0)
Smoking status									
* Do not smoke now*	28.8 (3.0)	29.2 (3.3)	28.7 (2.9)	26.9 (4.5)		11.9 (4.8)	9.5 (4.0)	10.6 (3.8)	7.5 (6.3)
* Occasional*	29.7 (12.7)	29.9 (13.8)	29.4 (12.2)	27.6 (18.7)		13.2 (23.4)	10.6 (19.1)	11.1 (18.4)	8.0 (30.3)
* Current-regular*	29.1 (6.0)	29.6 (6.5)	29.0 (5.8)	27.5 (8.9)		12.4 (9.5)	9.5 (7.8)	10.7 (7.5)	7.0 (12.3)
Heating duration per week									
* 0 hour*	29.3 (4.1)	29.9 (4.4)	29.3 (3.9)	27.5 (6.0)		11.8 (7.1)	9.6 (5.8)	9.9 (5.5)	7.8 (9.1)
* ≤84 hours*	28.6 (3.5)	28.9 (3.8)	28.4 (3.4)	26.7 (5.2)		12.2 (5.7)	9.5 (4.7)	11.1 (4.4)	6.9 (7.4)
* >84 hours*	29.1 (6.0)	29.7 (6.5)	29.2 (5.8)	27.6 (8.9)		12.2 (10.2)	9.6 (8.3)	10.6 (7.9)	8.0 (13.2)
Smoky house while heating									
* No*	28.8 (3.5)	29.2 (3.8)	28.6 (3.3)	26.8 (5.2)		12.3 (5.4)	9.8 (4.4)	11.3 (4.2)	7.5 (7.0)
* Yes*	28.8 (6.1)	29.0 (6.6)	28.7 (5.9)	27.2 (9.1)		11.9 (9.4)	8.7 (7.7)	10.1 (7.3)	6.6 (12.2)
Heating fuel type									
* No heating*	29.3 (4.1)	29.9 (4.4)	29.3 (3.9)	27.5 (6.1)		11.9 (7.0)	9.6 (5.7)	10.2 (5.4)	7.9 (9.0)
* Clean fuels only*	29.1 (6.3)	29.9 (6.8)	29.1 (6.0)	27.9 (9.3)		12.6 (10.0)	9.6 (8.2)	11.2 (7.8)	8.2 (13.0)
* Solid fuels included*	28.6 (3.5)	28.8 (3.8)	28.5 (3.4)	26.6 (5.2)		12 (5.7)	9.4 (4.7)	10.8 (4.4)	6.8 (7.4)
Cooking frequency									
* Infrequent*	28.9 (5.9)	29.4 (6.4)	28.9 (5.7)	27.2 (8.7)		12.0 (8.8)	9.6 (7.2)	11.1 (6.9)	8.4 (11.4)
* Home daily*	29.2 (6.1)	29.3 (6.6)	29.1 (5.9)	27.4 (9.0)		11.5 (9.9)	9.5 (8.1)	9.7 (7.7)	6.7 (12.8)
* Personal daily*	28.9 (3.0)	29.3 (3.3)	28.7 (2.9)	26.9 (4.5)		12.3 (5.1)	9.5 (4.1)	10.8 (3.9)	7.2 (6.5)
Cooking fuel type									
* No cooking*	29.0 (4.2)	29.5 (4.6)	28.8 (4.1)	27.2 (6.2)		12.1 (7.7)	9.3 (6.3)	10.5 (6.0)	7.3 (10.0)
* Clean fuels only*	28.9 (3.0)	29.2 (3.2)	28.8 (2.9)	27 (4.4)		12.1 (4.5)	9.6 (3.6)	10.9 (3.5)	7.5 (5.8)
* Solid fuels included*	29.0 (4.1)	29.4 (4.4)	28.9 (3.9)	27.1 (6)		11.9 (6.5)	9.6 (5.3)	10.3 (5.0)	7.2 (8.3)
Stove hours									
* 0 hour*	29.0 (4.5)	29.4 (4.9)	29.0 (4.3)	27.2 (6.7)		11.6 (7.0)	9.5 (5.7)	10.4 (5.5)	7.5 (9.1)
* <3 hours*	28.9 (3.0)	29.3 (3.3)	28.7 (2.9)	27.0 (4.5)		12.3 (5.0)	9.6 (4.1)	10.9 (3.9)	7.3 (6.5)
* 4-6 hours*	28.4 (10.0)	29.0 (10.9)	28.3 (9.7)	26.9 (14.9)		12.1 (16.3)	9.1 (13.3)	10.1 (12.8)	7.4 (21.1)

**Table 3:** Performance of multiple linear regression and random forest models for personal temperature exposure prediction

	MLR model						RF model				
Model *	R^2^	RMSE	nRMSE	CV-R^2^	CV-RMSE		R^2^	RMSE	nRMSE	CV-R^2^	CV-RMSE
**Summer (n=391)** †											
* Basic*	0.371	2.901	0.197	0.375	2.912		0.955	0.775	0.053	0.880	1.283
* Basic+Questionnaire*	0.851	1.410	0.096	0.849	1.430		0.975	0.576	0.039	0.892	1.210
* Basic+Outdoor*	0.889	1.218	0.083	0.894	1.225		0.973	0.600	0.041	0.882	1.276
* Basic+Living room*	0.911	1.093	0.074	0.915	1.101		0.979	0.525	0.036	0.902	1.155
* Basic+Kitchen*	0.882	1.256	0.085	0.887	1.265		0.975	0.582	0.040	0.887	1.244
* Basic+Outdoor+Living room*	0.918	1.049	0.071	0.921	1.061		0.982	0.490	0.033	0.912	1.102
* Basic+Outdoor+Kitchen*	0.900	1.155	0.079	0.904	1.170		0.976	0.564	0.038	0.891	1.220
* Basic+Outdoor+Living room+Kitchen*	0.919	1.044	0.071	0.921	1.062		0.983	0.476	0.032	0.912	1.091
* Basic+Questionnaire+Outdoor+Living room+Kitchen*	0.923	1.015	0.069	0.922	1.051		0.983	0.478	0.033	0.917	1.070
**Winter (n=403)**											
* Basic*	0.418	2.789	0.128	0.422	2.798		0.840	1.463	0.067	0.604	2.343
* Basic+Questionnaire*	0.587	2.349	0.108	0.549	2.468		0.923	1.011	0.046	0.642	2.213
* Basic+Outdoor*	0.422	2.779	0.128	0.425	2.791		0.895	1.185	0.054	0.623	2.294
* Basic+Living room*	0.662	2.125	0.098	0.667	2.123		0.922	1.023	0.047	0.665	2.148
* Basic+Kitchen*	0.630	2.225	0.102	0.633	2.222		0.913	1.077	0.049	0.640	2.222
* Basic+Outdoor+Living room*	0.664	2.119	0.097	0.669	2.119		0.930	0.966	0.044	0.686	2.075
* Basic+Outdoor+Kitchen*	0.630	2.224	0.102	0.633	2.223		0.920	1.031	0.047	0.646	2.217
* Basic+Outdoor+Living room+Kitchen*	0.680	2.068	0.095	0.685	2.063		0.936	0.927	0.043	0.691	2.073
* Basic+Questionnaire+Outdoor+Living room+Kitchen*	0.709	1.970	0.090	0.683	2.063		0.928	0.981	0.045	0.699	2.018

* Predictors in “Basic” models included age, sex, and measurement date (as a natural cubic smooth function for the first date of measurement with 3 degrees of freedom); Extra predictors of “Basic+Questionnaire” models in summer included area, income, occupation, indoor time %, education, and dwelling type; “Basic+Questionnaire” models in winter included smoky house while heating, heating fuel type, area, heating duration per week, income, occupation, stove hours, cooking frequency, dwelling type, smoking status, indoor time %, kitchen window open, and household size.

† n: the number of participants included in the model.

Abbreviations: CV-R2, 10-fold cross-validated R-squared; CV-RMSE, 10-fold cross-validated root mean square error; MLR model, multiple linear regression model; nRMSE, normalized root mean square error; R2, R-squared; RF model, random forest model; RMSE, root mean square error.

## Data Availability

The China Kadoorie Biobank (CKB) is a global resource for the investigation of lifestyle, environmental, blood biochemical and genetic factors as determinants of common diseases. The CKB study group is committed to making the cohort data available to the scientific community in China, the UK and worldwide to advance knowledge about the causes, prevention and treatment of disease. For detailed information on what data is currently available to open access users and how to apply for it, visit: https://www.ckbiobank.org/data-access Researchers who are interested in obtaining the raw data from the China Kadoorie Biobank study that underlines this paper should contact ckbaccess@ndph.ox.ac.uk. A research proposal will be requested to ensure that any analysis is performed by bona fide researchers and - where data is not currently available to open access researchers - is restricted to the topic covered in this paper.
