# Supplementary material for: Modelling personal temperature exposure using household and outdoor temperature and questionnaire data: implications for epidemiological studies
Source: Environ Int. Author manuscript; Available in PMC 2024 Oct 28. (PMC7616742; doi:10.1016/j.envint.2024.109060)
Supplement: Supplementary file [file EMS199344-supplement-Supplementary_file.pdf]

Supplementary Appendix

Modelling personal temperature exposure using measured household and outdoor temperature and questionnaire data: implications for epidemiological studies

Xi Xia[1,2,3,4,5], Ka Hung Chan[3]*, Yue Niu[6], Cong Liu[6], Yitong Guo[3,5], Kin-Fai Ho[5], Steve Hung Lam Yim[7,8,9], Baihan Wang[3], Aiden Doherty[3,10,11], Daniel Avery[3], Pei Pei[12], Canqing Yu[12,13,14], Dianjianyi Sun[12,13,14], Jun Lv[12,13,14], Junshi Chen[15], Liming Li[12,13,14], Peng Wen[16], Shaowei Wu[1,2]*, Kin Bong Hubert Lam[3†], Haidong Kan[6,17†], Zhengming Chen[3†] on behalf of the China Kadoorie Biobank Study Group[#]

1 Department of Occupational and Environmental Health, School of Public Health, Xi'an Jiaotong University Health Science Center, Xi'an, China
2 Key Laboratory of Environment and Genes Related to Diseases, Ministry of Education, Xi'an, China
3 Clinical Trial Service Unit and Epidemiological Studies Unit, Nuffield Department of Population Health, University of Oxford, Oxford, UK
4 School of Public Health, Shaanxi University of Chinese Medicine, Xi'an, China
5 The Jockey Club School of Public Health and Primary Care, The Chinese University of Hong Kong, Hong Kong SAR, China
6 School of Public Health, Key Lab of Public Health Safety of the Ministry of Education and NHC Key Lab of Health Technology Assessment, Fudan University, Shanghai, China
7 Asian School of the Environment, Nanyang Technological University, Singapore
8 Lee Kong Chian School of Medicine, Nanyang Technological University, Singapore
9 Earth Observatory of Singapore, Nanyang Technological University, Singapore
10 Big Data Institute, Li Ka Shing Centre for Health Information and Discovery, University of Oxford, Oxford, UK
11 National Institute of Health Research Oxford Biomedical Research Centre, Oxford University Hospital NHS Foundation Trust, John Racliffe Hospital, Oxford, UK
12 Peking University Center for Public Health and Epidemic Preparedness & Response, Beijing, China
13 Department of Epidemiology and Biostatistics, School of Public Health, Peking University Health Science Center, Beijing, China
14 Key Laboratory of Epidemiology of Major Diseases (Peking University), Ministry of Education, Beijing, China
15 China National Center For Food Safety Risk Assessment, Beijing, China
16 Maiji Center for Disease Control and Prevention, Gansu, China
17 Children's Hospital of Fudan university, National Center for Children's Health, Shanghai, China

*Joint corresponding authors
[†]Joint senior authors
[#]Members of the CKB Collaborative Group are shown at the end of the manuscript

Address for correspondence
Ka Hung Chan: Big Data Institute, Old Road Campus, Roosevelt Drive, OX3 7LF; email: kahung.chan@ndph.ox.ac.uk
Shaowei Wu: Department of Occupational and Environmental Health, School of Public Health, Xi'an Jiaotong University Health Science Center, 76 Yanta West Road, Yanta District, Xi'an, Shaanxi 710061, China; email: shaowei_wu@xjtu.edu.cn

This supplement includes:

5 eTables and 5 eFigures

eTable 1. Categories of activity and location assessed in the time-activity questionnaire

Activity carried out	Location of activity
Cooking	Kitchen
Turn on heating at home	Living room
Turn off heating at home	Bedroom
Walk/ exercise	Home (unspecific)
Travel (cycle/ car/ bus)	Work/ public (indoors)
Travel (metro/ train)	Roadside
Work	Farm/ green space
Remove personal monitor	Work/ public (outdoors)
Put on personal monitor	Travel (cycle/ car/ bus)
Sleeping	Travel (metro/ train)
Sitting and none of the above	
Other	

eTable 2. Person-hour of temperature data across device location and season

	Overall (N=440) *		Summer (N=391)		Winter (N=403)	
	**Mean(SD)**	**Total**	**Mean(SD)**	**Total**	**Mean(SD)**	**Total**
Personal	198 (50.2)	87238	114 (17.4)	44467	106 (20.3)	42771
Living room	198 (50.2)	87238	114 (17.4)	44467	106 (20.3)	42771
Kitchen	198 (50.2)	87238	114 (17.4)	44467	106 (20.3)	42771
Outdoor	175 (56.9)	71413	112 (15.3)	40722	103 (20.4)	30691
Outdoor with ERA5 data †	200 (51.4)	88069	116 (18.4)	45450	106 (18.5)	42619

* The summer and winter samples overlap substantially, with 37 summer participants replaced by 35 other participants in the winter. † ERA5 data, regional temperature data derived from the well-established 5th generation ECMWF reanalysis database for global climate and weather based on the geolocation of the centroid of the study community.

eTable 3. Selected participant characteristics

Characteristics, n (%)	Overall (N=440) *	Summer (N=391)	Winter (N=403)	p value
Age, mean (SD), years	58.0 (6.8)	58.2 (6.6)	58.0 (6.8)	0.747
Female	320 (73%)	290 (74%)	298 (74%)	0.943
Education				0.998
No formal	110 (25%)	98 (25%)	101 (25%)	
Primary & middle school	157 (36%)	136 (35%)	141 (35%)	
High-school or above	173 (39%)	157 (40%)	161 (40%)	
Occupation				0.901
Agriculture & related workers	165 (38%)	144 (37%)	150 (37%)	
Factory worker	21 (4.8%)	21 (5.4%)	19 (4.7%)	
Home-maker	124 (28%)	105 (27%)	117 (29%)	
Non-manual labour	12 (2.7%)	9 (2.3%)	11 (2.7%)	
Self/un-employed or other	118 (27%)	112 (29%)	106 (26%)	
Income				0.665
<35,000 yuan	162 (37%)	138 (35%)	148 (37%)	
35,000-74,999 yuan	139 (32%)	133 (34%)	125 (31%)	
≥75,000 yuan	139 (32%)	120 (31%)	130 (32%)	
Household size >4	212 (48%)	191 (49%)	197 (49%)	0.992
Dwelling type				0.933
Apartment	16 (3.6%)	15 (3.8%)	15 (3.7%)	
House	424 (96%)	376 (96%)	388 (96%)	
Area				0.344
Gansu	146 (33%)	116 (30%)	139 (34%)	
Henan	147 (33%)	136 (35%)	132 (33%)	
Suzhou	147 (33%)	139 (36%)	132 (33%)	
Current-regular smoking	62 (14%)	49 (13%)	56 (14%)	0.634
Heating duration per week				0.619
0 hour	151 (34%)	140 (36%)	133 (33%)	
≤84 hours	54 (12%)	51 (13%)	50 (12%)	
>84 hours	235 (53%)	200 (51%)	220 (55%)	
Smoky house while heating	110 (25%)	85 (22%)	105 (26%)	0.349
Heating fuel type				0.697
No heating	151 (34%)	140 (36%)	133 (33%)	
Clean fuels only	52 (12%)	48 (12%)	50 (12%)	
Solid fuels included	237 (54)	203 (52)	220 (54)	
Cooking frequency				0.961
Infrequent	53 (12%)	44 (11%)	47 (12%)	
Home daily	52 (12%)	42 (11%)	45 (11%)	
Personal daily	335 (76%)	305 (78%)	311 (77%)	
Cooking fuel type				0.210
No cooking	102 (23%)	97 (25%)	79 (20%)	
Clean fuels only	201 (46%)	171 (44%)	189 (47%)	
Solid fuels included	137 (31%)	123 (31%)	135 (33%)	
Stove hours				0.927
0 hour	100 (23%)	81 (21%)	88 (22%)	
<3 hours	323 (73%)	295 (75%)	300 (74%)	
4-6 hours	17 (3.9%)	15 (3.8%)	15 (3.7%)	
Indoor time %, mean (SD) †	85.6 (12.0)	85.5 (12.0)	90.1 (10.0)	<0.001
At home time %, mean (SD) †	82.5 (11.3)	81.2 (11.8)	88.1 (10.4)	<0.001

* The summer and winter samples overlap substantially, with 37 summer participants replaced by 35 other participants in the winter. † Indoor time % was calculated from season-specific time-activity questionnaire.

eTable 4. Performance of generalised linear mixed effect models for personal temperature exposure prediction

Model *	R^2m	R^2c	RMSE	nRMSE	AIC	CV-R^2	CV-RMSE
**Summer (n=391, no. of data points=2318)** †							
Basic	0.717	0.913	1.102	0.053	8489	0.883	2.203
Basic+Questionnaire	0.787	0.901	1.145	0.056	8448	0.876	1.874
Basic+Outdoor	0.808	0.916	1.009	0.049	7875	0.900	1.675
Basic+Living room	0.864	0.934	0.934	0.045	7440	0.913	1.466
Basic+Kitchen	0.837	0.930	0.963	0.047	7664	0.906	1.604
Basic+Outdoor+Living room	0.875	0.938	0.895	0.043	7240	0.920	1.393
Basic+Outdoor+Kitchen	0.850	0.932	0.936	0.045	7505	0.912	1.517
Basic+Outdoor+Living room+Kitchen	0.877	0.939	0.892	0.043	7229	0.921	1.389
Basic+Questionnaire+Outdoor+Living room+Kitchen	0.878	0.938	0.888	0.043	7235	0.921	1.387
**Winter (n=403, no. of data points=2089)**							
Basic	0.495	0.794	1.787	0.057	9610	0.696	3.031
Basic+Questionnaire	0.534	0.826	1.711	0.055	9524	0.717	3.243
Basic+Outdoor	0.508	0.803	1.748	0.056	9542	0.712	2.994
Basic+Living room	0.637	0.831	1.613	0.052	9097	0.748	2.559
Basic+Kitchen	0.604	0.830	1.617	0.052	9163	0.745	2.681
Basic+Outdoor+Living room	0.636	0.832	1.606	0.052	9092	0.752	2.557
Basic+Outdoor+Kitchen	0.604	0.830	1.613	0.052	9163	0.748	2.677
Basic+Outdoor+Living room+Kitchen	0.644	0.835	1.584	0.051	9040	0.756	2.523
Basic+Questionnaire+Outdoor+Living room+Kitchen	0.657	0.849	1.534	0.049	8990	0.767	2.652

* Predictors in "Basic" model included participant as a random effect and age, sex, and measurement date (as a natural cubic smooth function for the first date of measurement with 3 degrees of freedom) as fixed effect terms; Extra predictors of "Basic+Questionnaire" model in summer included area, income, occupation, indoor time %, education, and dwelling type, for "Basic+Questionnaire" model in winter included smoky house while heating, heating fuel type, area, heating duration per week, income, occupation, stove hours, cooking frequency, dwelling type, smoking status, indoor time %, kitchen window open, and household size. † n: the number of participants included in the model; no. of data points: the number of data points included in the model.

Abbreviations: AIC, Akaike information criterion; CV-R^2, 10-fold cross-validated R-squared; CV-RMSE, 10-fold cross-validated root mean square error; nRMSE, normalized root mean square error; R^2c, conditional R-squared; R^2m, marginal R-squared; RMSE, root mean square error.

eTable 5. Performance of multiple linear regression and random forest models for personal temperature exposure prediction upon excluding ERA5 data

	MLR model					RF model				
**Model ***	**R^2**	**RMSE**	**nRMSE**	**CV-R^2**	**CV-RMSE**	**R^2**	**RMSE**	**nRMSE**	**CV-R^2**	**CV-RMSE**
**Summer (n=363) †**										
Basic	0.488	2.711	0.183	0.488	2.721	0.956	0.791	0.054	0.888	1.289
Basic+Questionnaire	0.865	1.393	0.094	0.858	1.436	0.976	0.582	0.039	0.896	1.225
Basic+Outdoor	0.890	1.255	0.085	0.894	1.264	0.974	0.613	0.041	0.883	1.318
Basic+Living room	0.916	1.100	0.074	0.918	1.106	0.980	0.541	0.037	0.908	1.162
Basic+Kitchen	0.886	1.278	0.086	0.887	1.286	0.975	0.597	0.040	0.895	1.235
Basic+Outdoor+Living room	0.920	1.072	0.073	0.922	1.078	0.982	0.506	0.034	0.912	1.139
Basic+Outdoor+Kitchen	0.902	1.187	0.080	0.904	1.197	0.977	0.568	0.038	0.894	1.261
Basic+Outdoor+Living room+Kitchen	0.921	1.066	0.072	0.922	1.074	0.983	0.496	0.034	0.914	1.126
Basic+Questionnaire+Outdoor+Living room+Kitchen	0.925	1.036	0.070	0.922	1.065	0.983	0.489	0.033	0.920	1.084
**Winter (n=299)**										
Basic	0.254	2.600	0.140	0.277	2.585	0.771	1.442	0.078	0.441	2.306
Basic+Questionnaire	0.482	2.167	0.117	0.430	2.289	0.894	0.982	0.053	0.515	2.112
Basic+Outdoor	0.314	2.494	0.134	0.336	2.469	0.872	1.076	0.058	0.448	2.249
Basic+Living room	0.628	1.836	0.099	0.637	1.806	0.904	0.931	0.050	0.559	1.986
Basic+Kitchen	0.560	1.997	0.107	0.587	1.964	0.890	0.998	0.054	0.511	2.146
Basic+Outdoor+Living room	0.630	1.831	0.099	0.637	1.804	0.913	0.887	0.048	0.587	1.942
Basic+Outdoor+Kitchen	0.566	1.984	0.107	0.589	1.954	0.895	0.974	0.052	0.520	2.115
Basic+Outdoor+Living room+Kitchen	0.648	1.787	0.096	0.658	1.761	0.921	0.846	0.046	0.617	1.881
Basic+Questionnaire+Outdoor+Living room+Kitchen	0.681	1.701	0.092	0.646	1.797	0.912	0.892	0.048	0.612	1.856

* Predictors in "Basic" models included age, sex, and measurement date (as a natural cubic smooth function for the first date of measurement with 3 degrees of freedom); Extra predictors of "Basic+Questionnaire" models in summer included area, income, occupation, indoor time %, education, and dwelling type; "Basic+Questionnaire" models in winter included smoky house while heating, heating fuel type, area, heating duration per week, income, occupation, stove hours, cooking frequency, dwelling type, smoking status, indoor time %, kitchen window open, and household size. † n: the number of participants included in the model.

Abbreviations: ERA5: 5th generation European centre for Medium-Range Weather Forecasts reanalysis database for global climate and weather; CV-R^2, 10-fold cross-validated R squared; CV-RMSE, 10-fold cross-validated root mean square error; MLR model, multiple linear regression model; nRMSE, normalized root mean square error; R^2, R-squared; RF model, random forest model; RMSE, root mean square error.

eFigure 1. Study areas of the China Kadoorie Biobank Cohort Study*

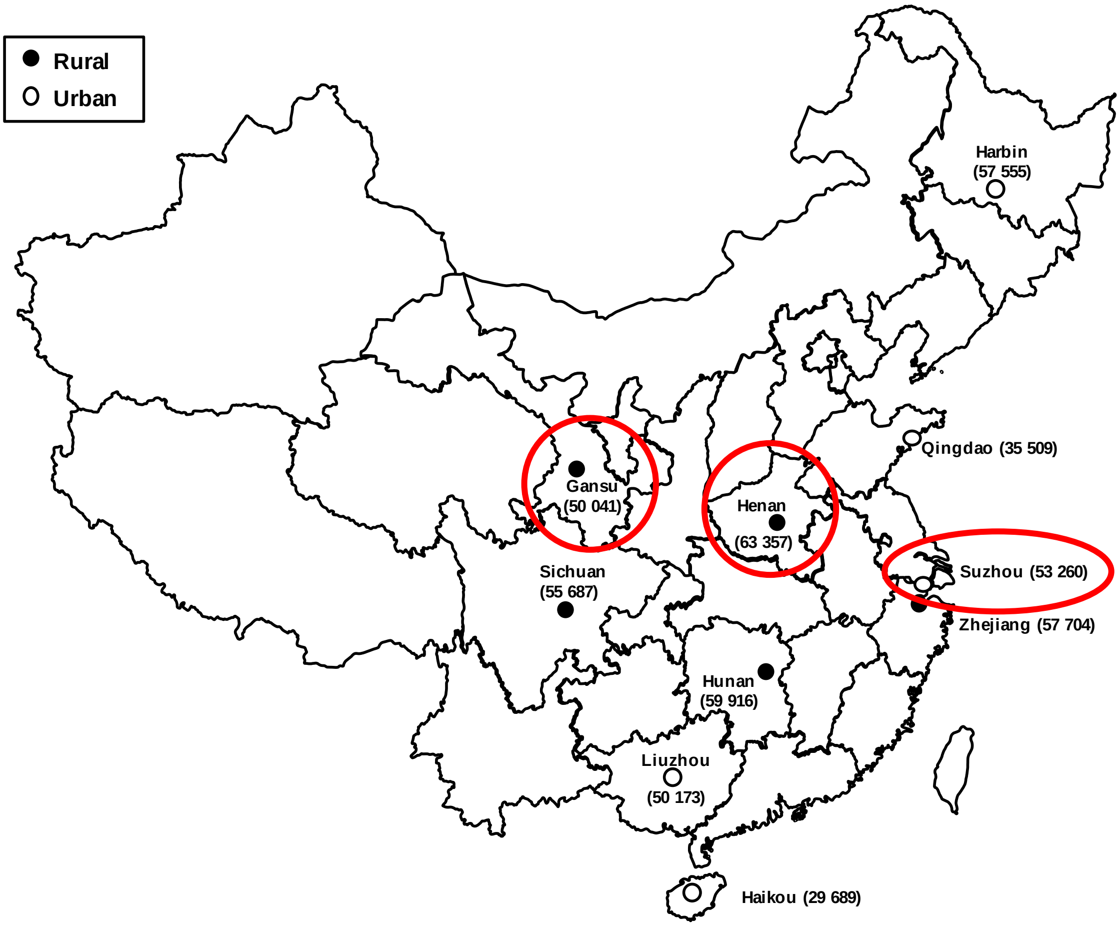

*Figure reproduced from Chen et al. Int J Epidemiol 2011. The figure illustrates the location of the ten study areas of the China Kadoorie Biobank Cohort Study, with black circles indicating the rural sites, and open circles indicating the urban sites. Numbers in brackets are the baseline sample size per study site. The one urban (Suzhou) and two rural (Gansu, Henan) sites included in the CKB-Air study are highlighted in red. This figure is for illustrative purpose and does not represent the exact geography of the wider region.

eFigure 2. Flow chart of data processing and participant exclusion

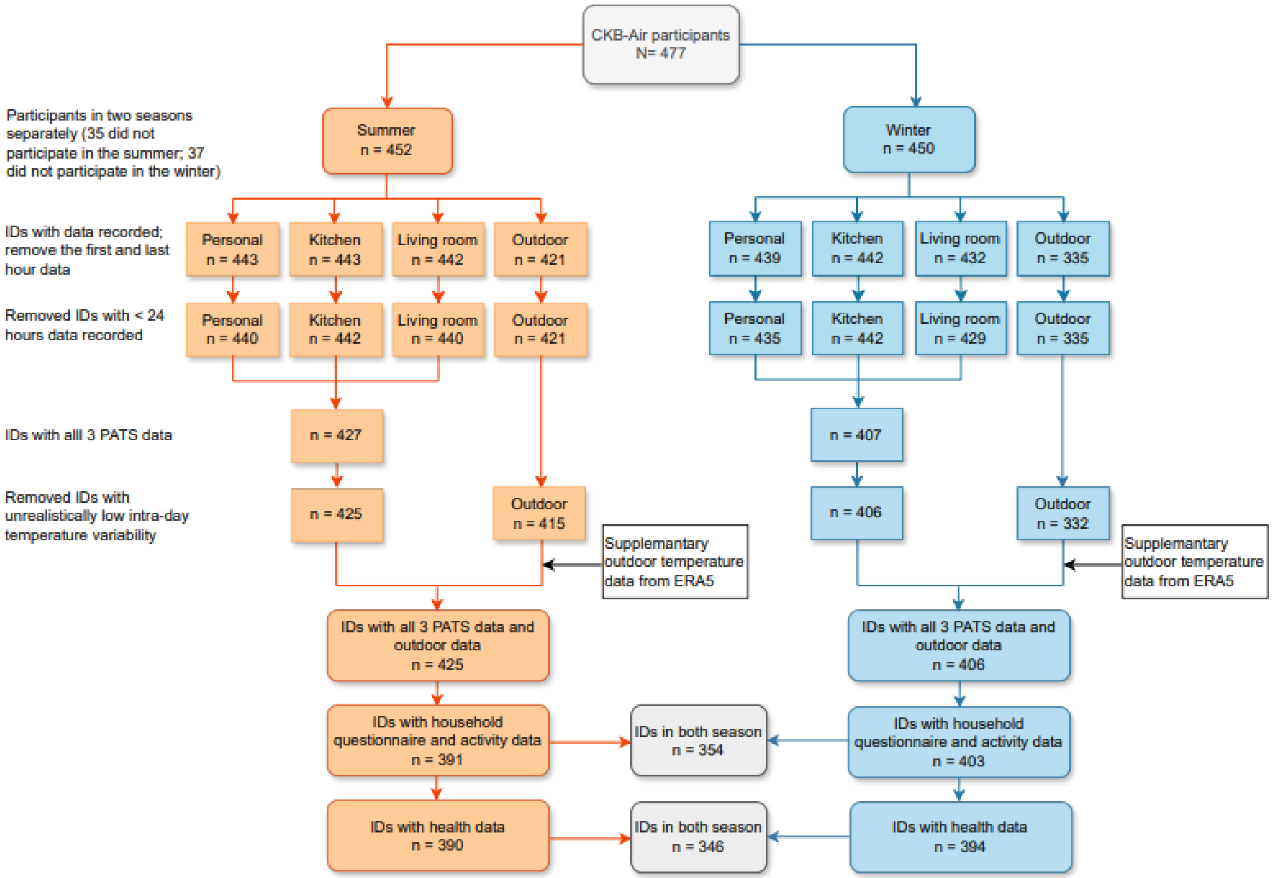

* ERA5 data, regional temperature data derived from the well-established 5th generation European centre for Medium-Range Weather Forecasts reanalysis database for global climate and weather based on the geolocation of the centroid of the study community.

eFigure 3. Intra-period and inter-season temperature variability*

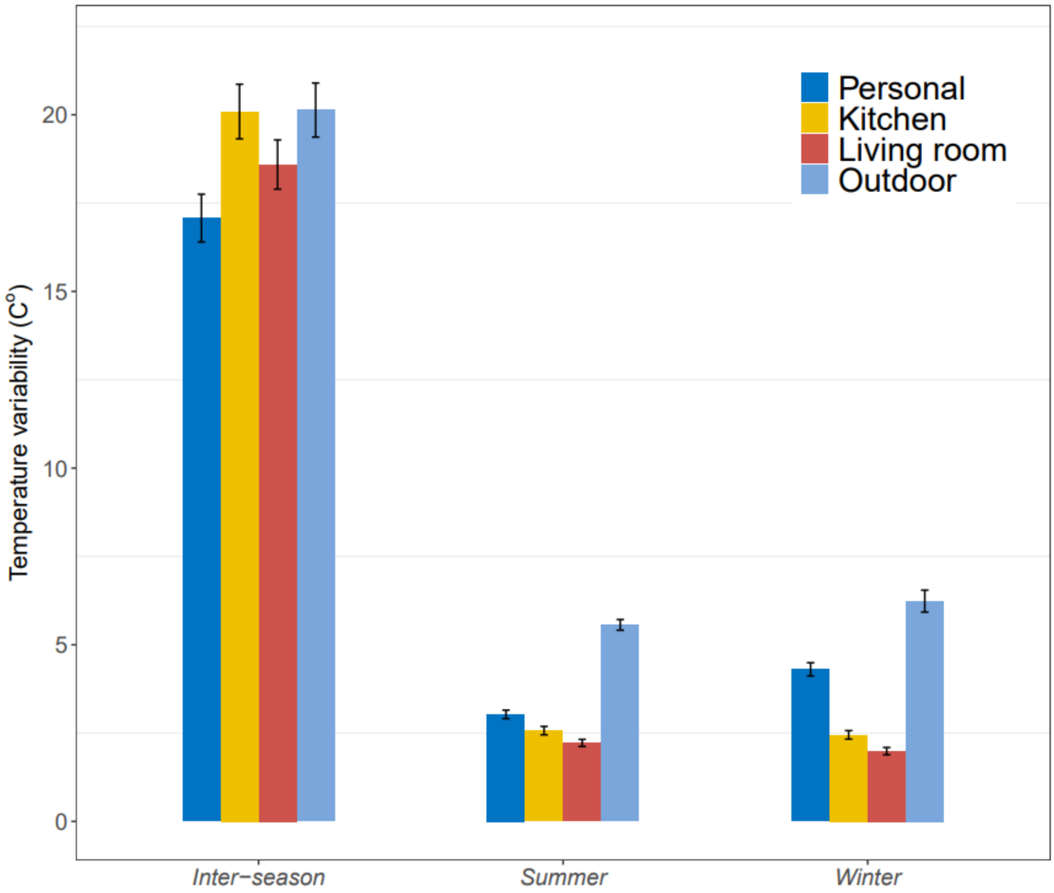

*Vertical black lines indicate the 95% confidence interval

eFigure 4. Spearman correlation matrix of 24-hour mean temperature by microenvironments and season

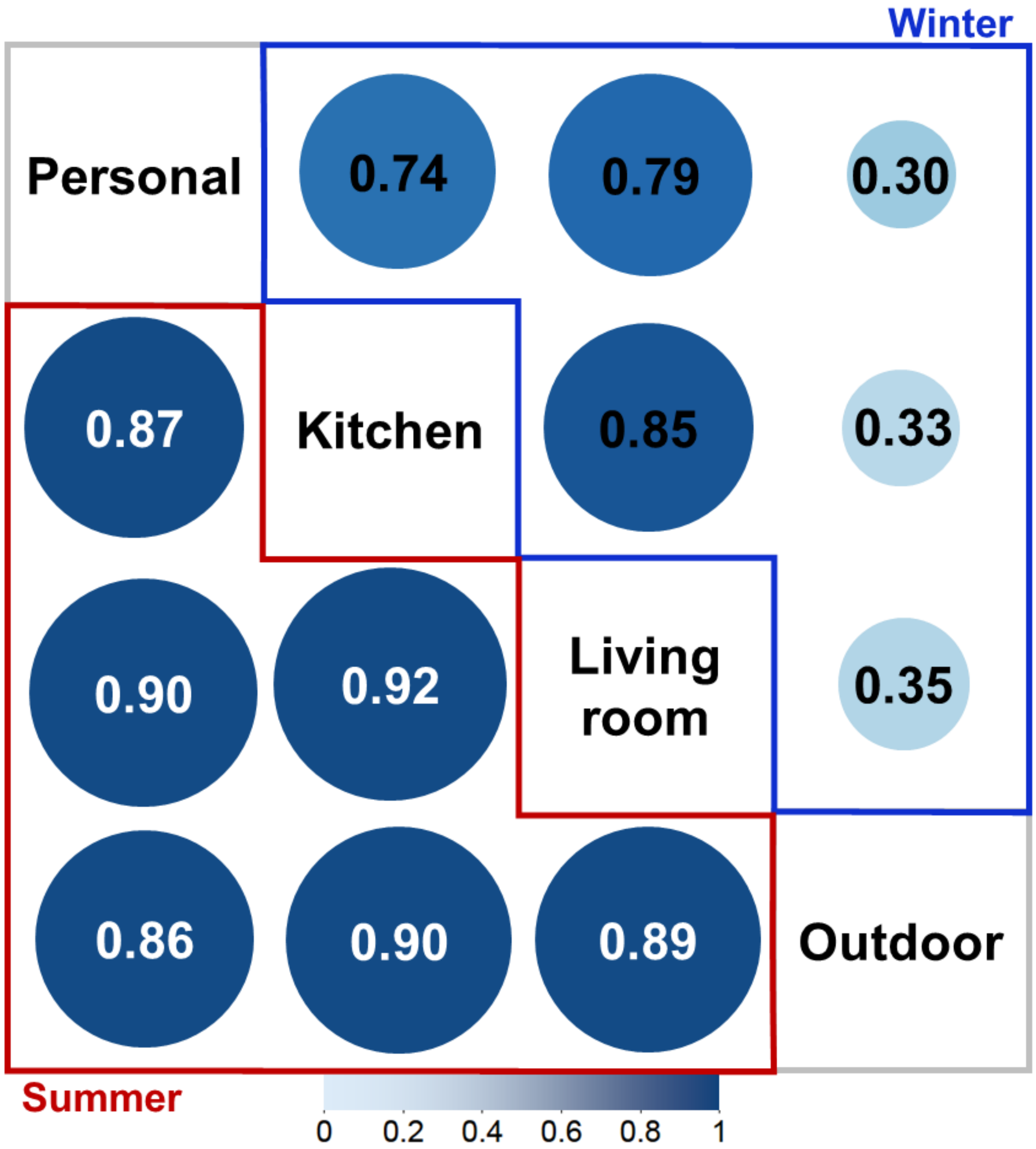

* The bottom-left cells with red backdrop correspond to data from summer; the top-right cells with light blue backdrop correspond to data from winter.

eFigure 5. Linear regression fitting plots of annual weighted average temperature across microenvironments

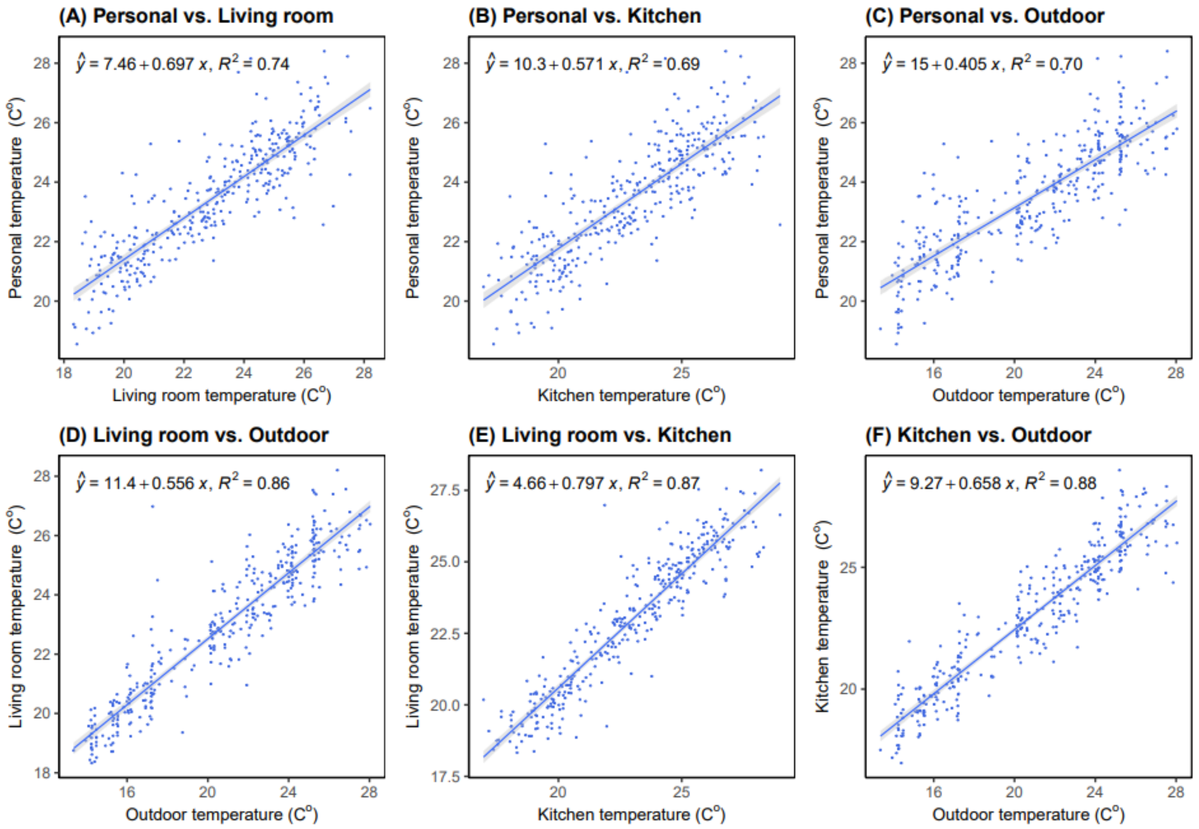

* Annual weighted average was calculated using the formula below:

$$Annual\ weighted\ average = \frac{\text{Temperature average in summer} \times 8 + \ \text{Temperature average in winter}\ \times 4}{12}$$
