# Supplementary figures and images for: Modelling personal temperature exposure using household and outdoor temperature and questionnaire data: implications for epidemiological studies
Source: Environ Int. Author manuscript; Available in PMC 2024 Oct 28. (PMC7616742; doi:10.1016/j.envint.2024.109060)

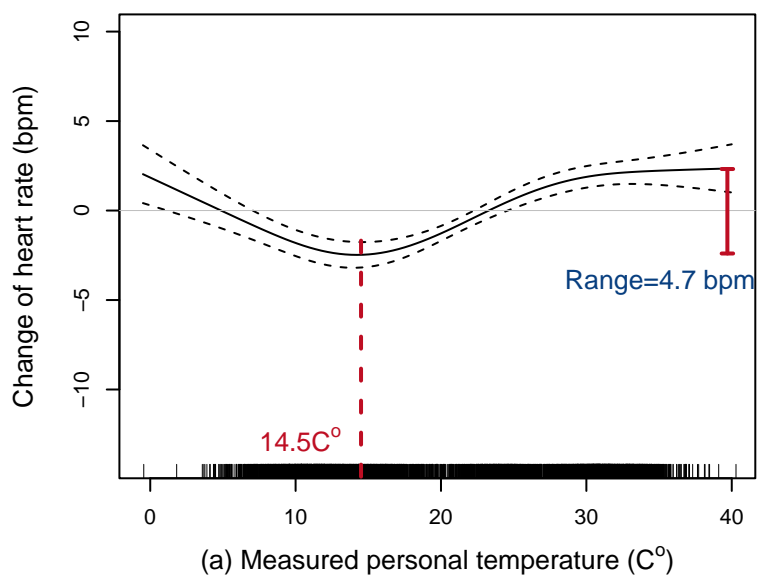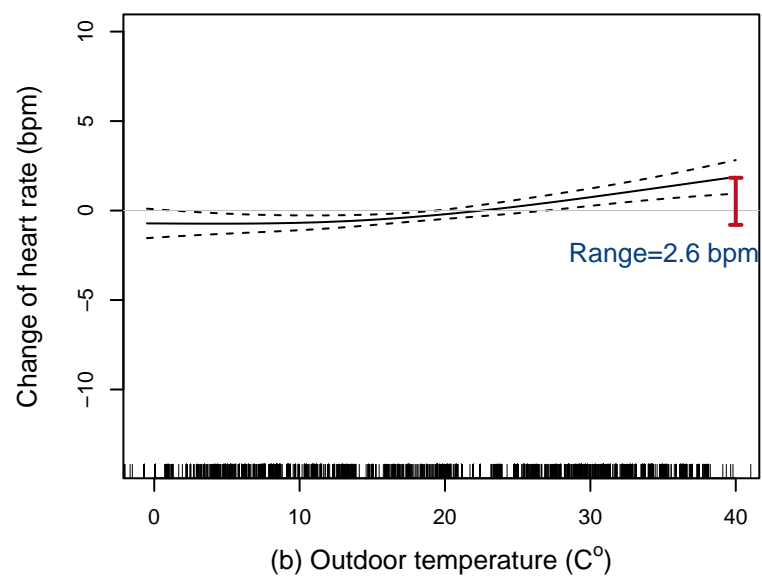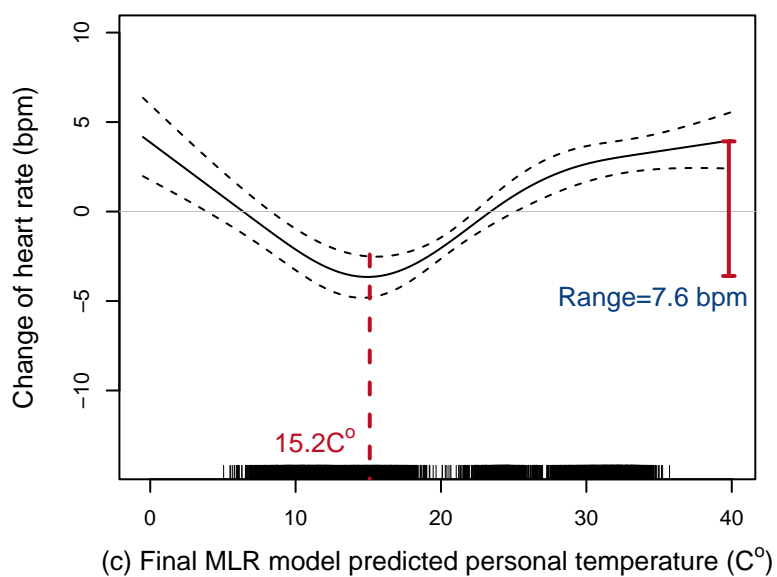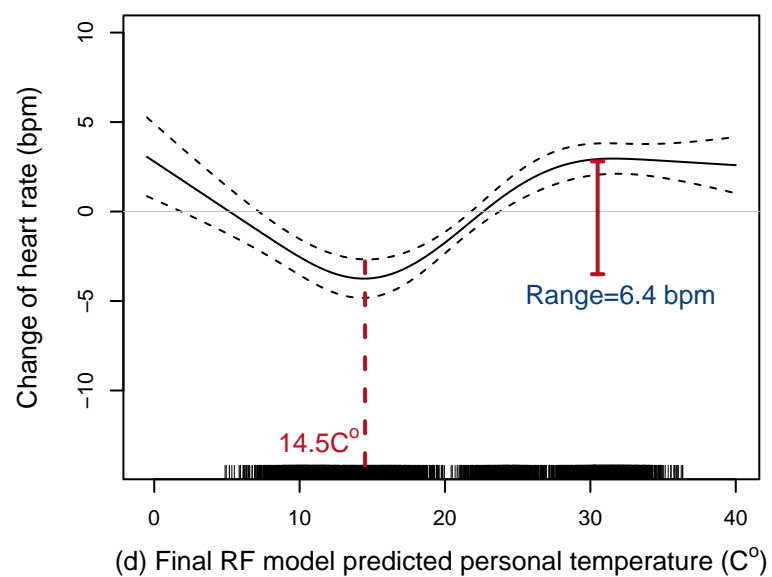

Supplement: Supplementary material [file EMS199344-supplement-Supplementary_material.pdf]
